# Parallel Streams of Direct Corticogeniculate Feedback from Mid-level Extrastriate Cortex in the Macaque Monkey

**DOI:** 10.1523/ENEURO.0364-23.2024

**Published:** 2024-03-13

**Authors:** Matthew Adusei, Edward M. Callaway, W. Martin Usrey, Farran Briggs

**Affiliations:** ^1^Neuroscience Graduate Program, University of Rochester, Rochester, New York 14642; ^2^Systems Neurobiology Laboratory, Salk Institute for Biological Sciences, La Jolla, California 92037; ^3^Center for Neuroscience, University of California Davis, Davis, California 95618; ^4^Department of Neurobiology, Physiology, and Behavior, University of California Davis, Davis, California 95616; ^5^Department of Neurology, University of California Davis, Davis, California 95618; ^6^Del Monte Institute for Neuroscience, University of Rochester School of Medicine and Dentistry, Rochester, New York 14642; ^7^Department of Neuroscience, University of Rochester School of Medicine and Dentistry, Rochester, New York 14642; ^8^Department of Brain and Cognitive Sciences, University of Rochester, Rochester, New York 14627; ^9^Center for Visual Science, University of Rochester, Rochester, New York 14627

**Keywords:** corticogeniculate, extrastriate, LGN, virus-mediated circuit tracing, visual cortex

## Abstract

First-order thalamic nuclei receive feedforward signals from peripheral receptors and relay these signals to primary sensory cortex. Primary sensory cortex, in turn, provides reciprocal feedback to first-order thalamus. Because the vast majority of sensory thalamocortical inputs target primary sensory cortex, their complementary corticothalamic neurons are assumed to be similarly restricted to primary sensory cortex. We upend this assumption by characterizing morphologically diverse neurons in multiple mid-level visual cortical areas of the primate (*Macaca mulatta*) brain that provide direct feedback to the primary visual thalamus, the dorsal lateral geniculate nucleus (LGN). Although the majority of geniculocortical neurons project to primary visual cortex (V1), a minority, located mainly in the koniocellular LGN layers, provide direct input to extrastriate visual cortex. These “V1-bypassing” projections may be implicated in blindsight. We hypothesized that geniculocortical inputs directly targeting extrastriate cortex should be complemented by reciprocal corticogeniculate circuits. Using virus-mediated circuit tracing, we discovered corticogeniculate neurons throughout three mid-level extrastriate areas: MT, MST, and V4. Quantitative morphological analyses revealed nonuniform distributions of unique cell types across areas. Many extrastriate corticogeniculate neurons had spiny stellate morphology, suggesting possible targeting of koniocellular LGN layers. Importantly though, multiple morphological types were observed across areas. Such morphological diversity could suggest parallel streams of V1-bypassing corticogeniculate feedback at multiple stages of the visual processing hierarchy. Furthermore, the presence of corticogeniculate neurons across visual cortex necessitates a reevaluation of the LGN as a hub for visual information rather than a simple relay.

## Significance Statement

First-order thalamic nuclei are most strongly connected in feedforward and feedback directions with primary sensory cortex. In the visual system, direct feedforward circuits link the visual thalamus with higher-order, extrastriate visual areas, although the function of these less common pathways remains unknown. Here we demonstrate for the first time the presence of complementary direct feedback connections between multiple extrastriate visual cortical areas and the visual thalamus in the primate brain. Although sparse, extrastriate corticogeniculate circuits are consistently present and contain morphologically diverse neurons, suggesting parallel streams of extrastriate feedback. These findings indicate an evolutionary conservation of circuits that integrate information from throughout the visual processing hierarchy within the visual thalamus.

## Introduction

Sensory information processing in the brain is often described as a feedforward progression from peripheral sensory receptors through subcortical structures to hierarchically organized cortical areas. For example, visual information is relayed from the retina to the dorsal lateral geniculate nucleus (LGN), to primary visual cortex (V1), before reaching extrastriate visual cortex (Felleman and Van Essen, 1991; Lamme et al., 1998). This focus on feedforward circuitry is due to strong anatomical, electrophysiological, and theoretical evidence that feedforward pathways must convey sensory signals rapidly to areas of the brain responsible for decisions and actions. Feedback circuits are present throughout sensory systems, but their role in sensory perception is modulatory and thus more challenging to decipher (Sherman and Guillery, 1998). When approaching sensory perception from the perspective of feedforward versus feedback pathways, it is important to ask if these circuits exist as loops rather than separate information processing pathways. Here, we provide striking new evidence that corticogeniculate circuits, the first feedback circuits in the visual system that connect visual cortex with the LGN (Sherman et al., 2006), are not limited to primary/secondary visual cortex in primates. Rather, reciprocal connections between the visual cortex and LGN are present throughout the visual cortical hierarchy. Though small in number, the presence of corticogeniculate neurons in all three visual area sampled suggests that these sparse connections are critical for vision and may play significant roles in residual visual perception when V1 is damaged.

Humans and nonhuman primates with damage to V1 can detect visual stimuli presented in the lesioned hemifield, a phenomenon known as blindsight (Sanders et al., 1974; Weiskrantz, 1996). Direct, “V1-bypassing” connections between the LGN and extrastriate cortex are thought to be critical for blindsight (Sincich et al., 2004; Ajina et al., 2015), although other structures like the superior colliculus and pulvinar may also contribute (Rodman et al., 1990; Warner et al., 2015). Interestingly, these extrastriate-projecting geniculocortical neurons are not evenly represented across the magnocellular, parvocellular, and koniocellular layers of the LGN. Most extrastriate-projecting geniculocortical neurons are found in the koniocellular layers (Bullier and Kennedy, 1983; Cowey and Stoerig, 1989; Rodman et al., 2001; Sincich et al., 2004). However, a recent study showed that following long-term V1 lesions, some MT-projecting geniculocortical neurons were found outside the koniocellular layers and expressed parvalbumin, a neurochemical marker for magnocellular and parvocellular LGN neurons, as well as calbindin, a marker for koniocellular neurons (Atapour et al., 2022). Surprisingly, while multiple studies have examined extrastriate-projecting geniculocortical neurons (Yukie and Iwai, 1981; Bullier and Kennedy, 1983; Cowey and Stoerig, 1989; Rodman et al., 2001; Sincich et al., 2004), none have investigated reciprocal, direct (i.e., V1-bypassing) corticogeniculate neurons in mid-level extrastriate cortex. The assumption being that corticogeniculate neurons, and corticothalamic neurons projecting to first-order thalamus in general, are present only in primary sensory cortices. If direct extrastriate corticogeniculate neurons exist, do these feedback circuits show a similar bias toward the koniocellular stream as their feedforward geniculocortical counterparts?

Here, we first asked whether mid-level extrastriate corticogeniculate neurons are present in the primate brain. We then sought evidence for parallel streams of extrastriate corticogeniculate neurons, perhaps with a bias toward the koniocellular stream. We reconstructed the complete dendritic morphologies of a large sample of virus-labeled corticogeniculate neurons in three mid-level extrastriate areas: middle temporal (MT), medial superior temporal (MST) and V4, of macaque monkeys. Quantitative analysis of extrastriate corticogeniculate morphology revealed the presence of multiple distinct neuronal subtypes with striking similarities to those observed previously in V1 (Briggs et al., 2016). Interestingly, the majority of corticogeniculate neurons in each extrastriate area were spiny stellate cells, suggesting a putative association with the koniocellular stream. Unbiased clustering analyses revealed a variety of cell- and area-specific projections, with distinct cell types from a single area dominating individual clusters. Overall, our findings suggest that direct reciprocal connectivity between the LGN and visual cortex is the rule; that is, connections to first-order thalamus are not limited to primary sensory cortex. Extrastriate corticogeniculate neurons are therefore positioned to influence signals transmitted through the LGN and could be critical for blindsight.

## Materials and Methods

The tissues examined for this study were prepared as part of a previous study (Briggs et al., 2016) and as a part of a separate optogenetics and neurophysiology study. All experimental methods using animals have been previously described in detail (Briggs et al., 2016) and were approved by the Animal Care and Use Committees of Dartmouth College, the University of California, Davis, and the Salk Institute for Biological Studies. Instructions for stereotactic injection of SADDG-EGFP/ChR2-mCherry rabies virus into the dorsal LGN, brain tissue harvesting, sectioning, staining, and reconstruction of virus-labeled neurons have also been previously detailed (Briggs et al., 2016; Bragg et al., 2017; Bragg and Briggs, 2017; Hasse et al., 2019) and are briefly described below.

Data from three adult macaques (*Macaca mulatta*), two male and one female, were analyzed for this study. To selectively label corticogeniculate neurons via retrograde infection, a genetically modified rabies virus, carrying genes encoding either enhanced green fluorescent protein or channelrhodopsin2 and m-Cherry (SADΔG-EGFP or -ChR2_mCherry, based on the SAD-B19 strain; Wickersham et al., 2007; Osakada et al., 2011) was injected into one hemisphere of the LGN (Briggs et al., 2016). In sterile surgery using aseptic techniques, modified rabies virus was injected into the LGN through a small craniotomy, under stereotaxic and neurophysiological guidance, using an injection pipette (glass pipette or Hamilton syringe). A total volume of 30 µl of virus was injected into a single LGN in Monkey 1 over six separate injection sites (targeting LGN layers 1–6), a total volume of 10 µl of virus was injected into a single LGN of Monkey 2 over two separate injection sites (targeting LGN layers 2–6); and a total volume of 5 µl of virus was injected into a single LGN in Monkey 3 over four separate injection sites (targeting LGN layers 1, 2, 4, and 5). We confirmed that virus injections were restricted to the LGN in all three monkeys [see Figure S1A of Briggs et al. (2016) for example of coronal LGN sections and 3D reconstructed injection site volumes within the LGN from Monkeys 1 and 3]. Injected virus did not fill the entire LGN but covered similar regions of the LGN in each monkey. Injections spanned all layers of the LGN and covered multiple retinotopic eccentricities, although the total volume of virus infection was larger in Monkey 1 [Briggs et al. (2016), their Fig. S1A]. Animals were allowed to recover for 7–11 days and were subsequently killed and perfused transcardially to collect brain tissue. The visual cortex was coronally sectioned at a thickness of 50–70 µm per section using a freezing microtome. All sections were stained for cytochrome oxidase activity to visualize subcortical structures and cortical layers. Sections were then labeled with a primary anti-GFP or anti-mCherry antibody (rabbit anti-GFP, Molecular Probes/Life Technologies; rabbit anti-DS red, Clontech Laboratories) followed by a biotinylated secondary antibody (goat anti-rabbit, Vector Laboratories) to facilitate avidin/biotin and DAB/peroxidase reactivity to permanently stain all virus-labeled corticogeniculate neurons. Sections were then mounted on glass slides, dehydrated, defatted, and coverslipped.

Three mid-level extrastriate visual cortical areas were examined for the presence of virus-labeled corticogeniculate neurons: the MT area, MST area, and area V4. Areal boundaries for each of these visual areas per section were identified by matching cytochrome oxidase-stained coronal brain tissue sections with a coronal-view macaque brain atlas (Paxinos et al., 2000). Areal boundaries per section were drawn using a Neurolucida system (MicroBrightField) with an Optronics camera attached to an Olympus Provis microscope (Olympus Corporation). Entire coronal tissue sections were traced, areal boundaries marked, and virus-labeled corticogeniculate neurons marked and counted using lower magnification (4× objective). Layer boundaries around reconstructed corticogeniculate neurons were traced at 4–10× based on cytochrome oxidase staining of the home section (containing the cell body). Because the boundaries between layers could be difficult to discern from cytochrome oxidase staining in some of extrastriate cortex, we computed the relative depths of each cortical layer in MT, MST, and V4 based on our cytochrome oxidase-stained tissue and compared that with depths of each layer per area from Nissl-stained tissue (from Brainmaps.org and University of Rochester Neuroscience Department anatomical archive). In all cases, our cytochrome oxidase-based laminar depths were within 2.5% of the laminar depths determined from Nissl-stained tissue. Individual neuronal dendritic morphologies were reconstructed under higher magnification (20–40×). Virus-labeled extrastriate corticogeniculate neurons were verified to have their cell bodies located in layer 6, consistent with prior observations of corticogeniculate neurons in V1/area 17 and V2/area 18 in primates and carnivores (Gilbert and Kelly, 1975; Lund et al., 1975; Lin and Kaas, 1977; Katz, 1987; Fitzpatrick et al., 1994; Usrey and Fitzpatrick, 1996; Ichida et al., 2014; Briggs et al., 2016; Hasse and Briggs, 2017a; Hasse et al., 2019; Adusei et al., 2021). Only six (four in MT and two in MST) out of ∼4,000 virus-labeled corticogeniculate neurons across all three monkeys appeared to have cell bodies displaced outside of layer 6. These neurons were all in regions of cortical curvature, and their home sections had darker cytochrome oxidase staining, making it difficult to discern laminar boundaries. These possibly displaced corticogeniculate neurons were not reconstructed.

Individual extrastriate corticogeniculate neurons were selected for reconstruction if they were well labeled, had their cell body entirely within a single tissue section (the home section), and were reasonably isolated from other labeled corticogeniculate neurons such that all dendritic processes could be unambiguously identified and attributed to the same neuron. Requiring cell bodies to be entirely within the home section enabled consistent estimation of cell body area. Reconstructed neurons were selected from distinct regions (e.g., from different eccentricities) of each area to ensure sampling of corticogeniculate neurons throughout each area. Neurons that could not be unambiguously assigned to MT, MST, or V4 based on their locations relative to sulci/gyri per section were not included in these analyses. A total of 150 corticogeniculate neurons (60 from V4; 50 from MT; and 40 from MST) were reconstructed from three animals: 75 corticogeniculate neurons from Monkey 1 (25 in MT, 25 in MST, 25 in V4); 55 corticogeniculate neurons from Monkey 2 (25 in MT, 15 in MST and 15 in V4); and 20 corticogeniculate neurons from Monkey 3 (all in V4). Although axons were sometimes visible and traceable, axonal information was excluded from subsequent morphological analyses since it was not possible to trace axons for the majority of labeled corticogeniculate neurons. Basal and apical dendritic arbors originating from the cell body were traced by placing nodes at each branch point and endings at each branch termination. For all neurons, dendritic arbors were followed through five adjacent sections: the home section containing the cell body and two sections immediately above and below the home section. In no case were additional dendrites found beyond the five adjacent sections examined. Dendritic spines of virus-labeled corticogeniculate neurons were not reconstructed even though they were visible in all corticogeniculate neurons.

Clustering algorithms, principal components analysis (PCA), and statistical methods were used to evaluate morphological differences between reconstructed corticogeniculate neurons, as described previously (Briggs et al., 2016; Bragg et al., 2017; Bragg and Briggs, 2017; Hasse et al., 2019; Adusei et al., 2021). Neurolucida Explorer (MicroBrightField) was used to extract the following morphological metrics from each reconstructed corticogeniculate neuron. Cell body area was computed as the area within the contour of the cell body, drawn through the largest extent of the cell body in the home section. The total number of nodes on all dendrites—apical and basal—was extracted for each reconstructed neuron. Cell body position within layer 6 was measured as the proportional distance between the cell body and the contour drawn to represent the top of layer 6 compared with the full extent of layer 6 (measured as the distance between the top of layer 6 and the layer 6/white matter border). The height of the apical dendrite was measured as the proportional distance between the top of the apical dendrite and the top of layer 2/3 compared with the full cortical depth (measured as the distance between the top of layer 2/3 and the layer 6/white matter border). For spiny stellate neurons that lacked apical dendrites, the end of the dendrite closest to the top of layer 2/3 was used for an analogous measurement of tallest point on the dendritic tree. Percentages of basal dendrite in layers 4, 5, and 6 and in the white matter were calculated by dividing the basal dendrite per layer by the total length of basal dendrite per neuron and converting the resulting proportion to percentage. Percentages of apical dendrite in layers 2/3, 4, 5, and 6 and above layer 2/3 represented the percentage conversion of the ratio of the apical dendrite in each layer divided by the total length of the apical dendrite per neuron.

A cluster analysis was performed on all 150 reconstructed neurons to define morphologically distinct clusters of corticogeniculate neurons. There were no differences in morphology per cell type across monkeys, so data from all monkeys were combined for the cluster analysis and all subsequent analyses. Importantly, no information about the area of origin (MT, MST, or V4) was included in the cluster analysis. The cluster analysis was run using the following 11 independent morphological metrics: cell body area (which was *z*-scored due to the wider range in values compared with other metrics); percentage of basal dendrite in layers 4, 5, and 6 and the white matter; percentage of apical dendrite in layers 2/3, 4, 5, and 6; cell body position in layer 6; and apical dendrite height or tallest point on spiny stellate neurons (Tables 1–3). Metrics for which only a small proportion of corticogeniculate neurons had nonzero values were excluded from the cluster analysis. For example, if <10% of corticogeniculate neurons had >1% of their dendrites in a given layer, percentage of dendrite in this layer was not included in the cluster analysis. Based on these criteria, percentage of apical dendrite above layer 2/3 was excluded from the cluster analysis. Notably, since each metric is independently weighted by the clustering algorithm (Thorndike, 1953; Cauli et al., 2000; Bragg et al., 2017), removing or including this metric did not result in a redistribution of clusters. Additionally, cluster assignments were the same whether cell body area was *z*-scored or not. For the cluster analysis, the Euclidean distance between each neuron, defined by a point in an 11-dimensional space, was computed using the “pdist” function in Matlab (MathWorks). Clusters were defined by the inner squared distance between neurons using the “linkage” function and applying Ward's method. The “dendrogram” function was used to visualize the linkage distances between neurons and to illustrate clusters (Fig. 2*A*).

**Table 1.** Average ± SEM for three morphological metrics included in the cluster analysis: cell body (CB) area (*z*-scored), cell body position as percent depth in layer 6 (L6) where larger values indicate deeper position closer to the white matter, and height of the apical dendrite (AD) as percent depth in the cortical layers where smaller values indicate shallower position closer to layer 1

	CB area (µm^2^)	CB position in L6	AD height
Cluster 1 (*n *= 23)	0.36 ± 0.21	41.0 ± 4.2	76.9 ± 1.4
Cluster 2 (*n *= 22)	0.64 ± 0.29	19.3 ± 2.1	63.7 ± 2.8
Cluster 3 (*n *= 22)	0.03 ± 0.23	21.5 ± 2.6	24.0 ± 3.0
Cluster 4 (*n *= 7)	−0.14 ± 0.21	11.1 ± 1.3	46.2 ± 3.6
Cluster 5 (*n *= 25)	0.08 ± 0.16	18.8 ± 2.5	7.6 ± 1.7
Cluster 6 (*n *= 13)	−0.76 ± 0.10	59.0 ± 2.0	6.8 ± 0.8
Cluster 7 (*n *= 24)	−0.31 ± 0.16	69.8 ± 3.2	34.8 ± 3.2
Cluster 8 (*n *= 14)	−0.48 ± 0.15	50.7 ± 6.3	33.3 ± 4.3
*p* values	5.8 × 10^−5^	1.2 × 10^−17^	1.2 × 10^−23^
Differences	1,2,3,5 > 6	1 > 2,4,5; 6,7,8 > 2,3,4,5	1 > 3,5,6,7,8; 2 > 3,5,6,8; 4,7 > 5,6

*p* values for statistical differences across clusters are Bonferroni corrected for multiple comparisons at α = 0.0045; Significant differences between clusters are also listed below.

**Table 2.** Average ± SEM for four morphological metrics included in the cluster analysis: percent of apical dendrite (AD) in layer 6 (L6), layer 5 (L5), layer 4 (L4), and layer 2/3 (L2/3)

	AD in L6	AD in L5	AD in L4	AD in L2/3
Cluster 1 (*n *= 23)	0	0	0	0
Cluster 2 (*n *= 22)	0	0	0	0
Cluster 3 (*n *= 22)	14.2 ± 2.2	22.9 ± 2.4	45.4 ± 2.6	17.6 ± 2.2
Cluster 4 (*n *= 7)	15.9 ± 3.0	48.2 ± 2.0	35.2 ± 4.0	0.7 ± 0.6
Cluster 5 (*n *= 25)	7.4 ± 1.3	11.3 ± 1.2	20.9 ± 1.6	60.4 ± 2.5
Cluster 6 (*n *= 13)	28.3 ± 2.6	12.5 ± 1.3	22.9 ± 3.2	36.4 ± 4.9
Cluster 7 (*n *= 24)	40.0 ± 3.3	19.0 ± 2.8	27.0 ± 2.6	9.9 ± 2.5
Cluster 8 (*n *= 14)	66.3 ± 3.9	15.6 ± 2.6	11.3 ± 1.5	6.8 ± 2.8
*p* values	6.9 × 10^−25^	3.4 × 10^−21^	2.1 × 10^−22^	5.6 × 10^−23^
Differences	3,4,5,6 > 1,2; 7,8 > 1,2,3,5	3,5,6,7,8 > 1,2; 4 > 1,2,5,6	4,5,6,7,8 > 1,2; 3 > 1,2,5,7,8	3,8 > 1,2; 5 > 1,2,3,4,7,8; 6 > 1,2,4,7

Conventions as in Table 1.

**Table 3.** Average ± SEM for four morphological metrics included in the cluster analysis: percent of basal dendrite (BD) in the white matter (WM), and in layer 6 (L6), layer 5 (L5), and layer 4 (L4)

	BD in WM	BD in L6	BD in L5	BD in L4
Cluster 1 (*n *= 23)	0.4 ± 0.4	98.4 ± 0.8	1.2 ± 0.5	0
Cluster 2 (*n *= 22)	0.4 ± 0.3	79.4 ± 2.8	20.3 ± 2.8	0
Cluster 3 (*n *= 22)	0.1 ± 0.1	91.4 ± 2.1	7.9 ± 1.9	0.6 ± 0.5
Cluster 4 (*n *= 7)	1.8 ± 1.6	91.5 ± 3.0	6.7 ± 3.1	0
Cluster 5 (*n *= 25)	0	91.0 ± 2.5	6.0 ± 1.7	3.0 ± 1.1
Cluster 6 (*n *= 13)	2.2 ± 0.8	96.1 ± 1.3	1.1 ± 0.7	0.5 ± 0.4
Cluster 7 (*n *= 24)	2.8 ± 1.5	91.5 ± 1.8	4.5 ± 1.0	1.2 ± 0.7
Cluster 8 (*n *= 14)	0.7 ± 0.4	97.3 ± 1.4	1.7 ± 0.9	0.3 ± 0.3
*p* values	5.0 × 10^−6^	1.5 × 10^−7^	5.7 × 10^−10^	1.7 × 10^−5^
Differences	6 > 1,2,3,5; 8 > 1,5; 7 > 5	1 > 2,3,5; 5,6,7 > 2	2 > 1,3,5,6,7,8; 3 > 1,6	5 > 1,2,3,4

Conventions as in Table 1.

Two independent tests were performed to statistically verify the optimal number of clusters of distinct extrastriate corticogeniculate neuronal types. First, the Calinski/Harabasz method was applied to evaluate the optimal number of clusters based on a defined set of criteria, using the “evalclusters” function (Calinski and Harabasz, 1974). This test determined the optimal cluster number between 1 and 8 possible clusters. Next, Gaussian mixture models (GMMs) were used to independently test optimal cluster number (Talebi and Baker, 2016). Using the same 11 morphological metrics applied in the cluster analysis, a PCA was performed (Fig. 2*B*), and the PCA scores were used in the GMMs, again assuming 1–8 possible clusters (using the “fitgmdist” Matlab function). Three different GMM evaluations were then tested: negative log likelihood, Akaike information criterion, and Bayes information criterion. The GMM with the lowest criteria across the three evaluations indicated the optimal number of clusters.

Having determined the optimal number of clusters using the statistical methods described above, all reconstructed corticogeniculate neurons were then grouped according to their cluster assignment. The number of corticogeniculate neurons originating in each extrastriate area per cluster was determined. Statistical tests were then performed to assess possible differences in morphological metrics across clusters using nonparametric multiple-comparisons tests (Kruskal–Wallis one-way ANOVA) with *p* values corrected for multiple comparisons (11 comparisons/metrics) using Bonferroni’s correction given α = 0.0045. Averages and standard error on the mean (SEM), along with *p* values and differences among clusters for each metric, are reported in Tables 1–3.

## Results

Direct, V1-bypassing geniculocortical inputs to extrastriate visual cortex have been documented (Bullier and Kennedy, 1983; Cowey and Stoerig, 1989; Rodman et al., 2001; Sincich et al., 2004). However, reciprocal corticogeniculate circuits that similarly connect extrastriate visual cortex with the LGN directly, also bypassing V1, have never been confirmed using monosynaptic tracing methods (but see Lin and Kaas, 1977). We sought evidence for reciprocal extrastriate corticogeniculate feedback connections that complement extrastriate-projecting geniculocortical neurons in the primate brain. We utilized a virus-mediated monosynaptic circuit tracing approach using a modified rabies virus, SADDG-EGFP or ChR2/mCherry, that lacks the gene encoding an essential glycoprotein (G-gene) required to cross synapses. SADDG exclusively infects neurons in a retrograde manner (Kelly and Strick, 2000; Wickersham et al., 2007; Ghanem and Conzelmann, 2016); thus, the only neurons in the visual cortex to express EGFP or mCherry are those with monosynaptic feedback connectivity with the LGN, that is, corticogeniculate neurons. Previously, we used rabies virus-mediated circuit tracing to characterize the morphology of corticogeniculate neurons in V1 and V2 of macaque monkeys (Briggs et al., 2016). Here, we searched for virus-infected corticogeniculate neurons in macaque mid-level extrastriate visual areas MT, MST, and V4 following SADDG-EGFP or ChR2/mCherry injection into the LGN.

**Figure 1.**
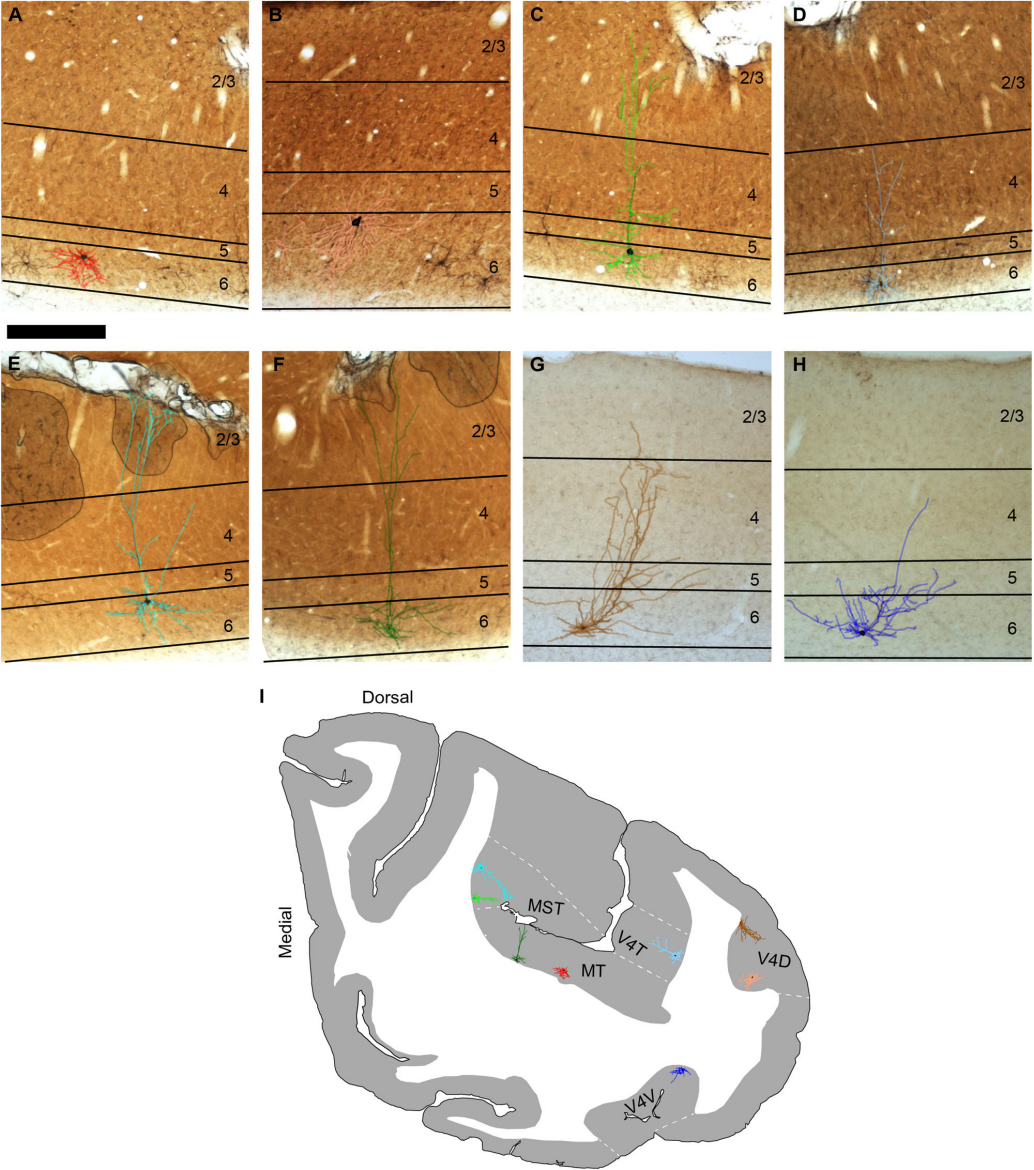
Virus-labeled corticogeniculate neurons originating in mid-level extrastriate areas MT, MST, and V4. ***A–H***, Photographs of coronal sections through area MT (***A***,***F***), MST (***C***,***E***), and V4 (***B***,***D***,***G***,***H***), showing representative examples of reconstructed virus-labeled corticogeniculate neurons. Scale bar is 750 µm and applies to all images. Colored reconstructions are overlaid onto photographs and indicate cluster assignment described in Figure 2. The same eight reconstructions are reproduced in Figure 2*B* to visualize the arbors more clearly. ***I***, Contour tracing of a coronal section showing relative locations of corticogeniculate neurons in ***A–H***. Medial is left and dorsal is up. Area boundaries are indicated by white dashed lines and areas are labeled. Note that the single representative coronal section tracing was not the home section for each corticogeniculate reconstruction, but a section chosen because all three areas of interest were represented.

Sparse populations of virus-labeled corticogeniculate neurons were consistently present in all three mid-level extrastriate visual areas (Fig. 1). Overall, we observed ∼4,000 virus-labeled corticogeniculate neurons in areas MT, MST, and V4 across 3 monkeys (∼3,200 in Monkey 1, ∼600 in Monkey 2, and ∼200 in Monkey 3). In all three monkeys, the number of virus-labeled corticogeniculate neurons in extrastriate areas was an order of magnitude less than the number of virus-labeled corticogeniculate neurons in V1 and V2. On average, there were ∼20 virus-labeled corticogeniculate neurons per section in V4, ∼10 per section in MST, and ∼30 per section in area MT. Notably, every section per area contained at least one virus-labeled corticogeniculate neuron. Labeled corticogeniculate neurons—especially those in MT—often formed clusters (Fig. 1*A*). Also, there were clear qualitative differences in the density of virus-labeled corticogeniculate neurons between the three extrastriate areas. Even though more sections contained V4, which is larger in area compared with MT and MST, MT appeared to have the highest density of labeled corticogeniculate neurons. MST had the smallest proportion of corticogeniculate neurons, possibly because there were fewer sections containing area MST. Consistent with previous observations in areas 17/V1, 18/V2, 21a, and posteromedial lateral suprasylvian area, posterolateral lateral suprasylvian area of carnivores and primates (Gilbert and Kelly, 1975; Lund et al., 1975; Lin and Kaas, 1977; Katz, 1987; Fitzpatrick et al., 1994; Usrey and Fitzpatrick, 1996; Ichida et al., 2014; Briggs et al., 2016; Hasse et al., 2019; Adusei et al., 2021), all but six virus-labeled cell bodies were spatially restricted to layer 6 (Fig. 1).

Because virus-mediated expression of EGFP or mCherry was extensive, labeling multiple corticogeniculate neurons per area, it was possible to reconstruct large samples of corticogeniculate neurons per area. Additionally, because fluorescent markers were expressed throughout dendritic arbors, we could reconstruct the full dendritic morphology of each corticogeniculate neuron, following dendrites through adjacent tissue sections (Fig. 1; see Materials and Methods). Altogether, the virus-mediated circuit tracing approach enabled us to apply rigorous statistical methods to classify distinct morphological subtypes within and across extrastriate visual areas and to specifically test whether extrastriate corticogeniculate neurons were more or less aligned with the koniocellular stream based on morphology. In other words, we asked whether there was an abundance of virus-labeled neurons with morphologies other than standard short and tall pyramidal morphologies that target parvocellular and magnocellular LGN, respectively. There were notable differences in dendritic morphology across extrastriate corticogeniculate neurons in areas MT, MST, and V4. Figure 1 shows reconstructions of the full dendritic arborizations of eight representative extrastriate corticogeniculate neurons. The relative locations of each of the eight cells in MT, MST, or V4 are shown in a representative coronal section tracing (Fig. 1*I*). Qualitative morphological assessments revealed that each of the three extrastriate areas examined contained all previously identified corticogeniculate cell types. These included spiny stellate corticogeniculate neurons (Fig. 1*A*,*B*), tall pyramidal corticogeniculate neurons with or without apical dendritic tufts in and above layer 2/3 (Fig. 1*C*,*E*,*F*), short corticogeniculate neurons with tilted or straight apical dendrites that terminate in layer 4 (Fig. 1*D*,*H*), as well as recently identified “intermediate” pyramidal cells with apical dendrite height intermediate to that of standard tall and short pyramidal corticogeniculate neurons (Fig. 1*G*; Gilbert and Kelly, 1975; Katz, 1987; Ichida et al., 2014; Briggs et al., 2016; Hasse et al., 2019; Adusei et al., 2021). Importantly, neurons that were qualitatively similar shared some dendritic features, like branched apical dendrites at similar laminar locations or extending basal dendrites with similar shapes, suggesting that extrastriate corticogeniculate neurons could form discrete groupings or clusters, explored further below. We also noted differences in the proportions of qualitatively defined cell types in the three areas. Area MT was heavily dominated by spiny stellate cells: 1,544 out of 2,032 or 76% of total virus-labeled corticogeniculate neurons in MT were spiny stellates. Almost two-thirds of the population of corticogeniculate neurons in MST also had spiny stellate morphology: 466 of 685 or 68% of MST corticogeniculate neurons. While most of the cells in V4 had spiny stellate morphology, this cell type was less dominant: 654 of 1,283 or 51% of V4 corticogeniculate neurons were spiny stellate cells.

**Figure 2.**
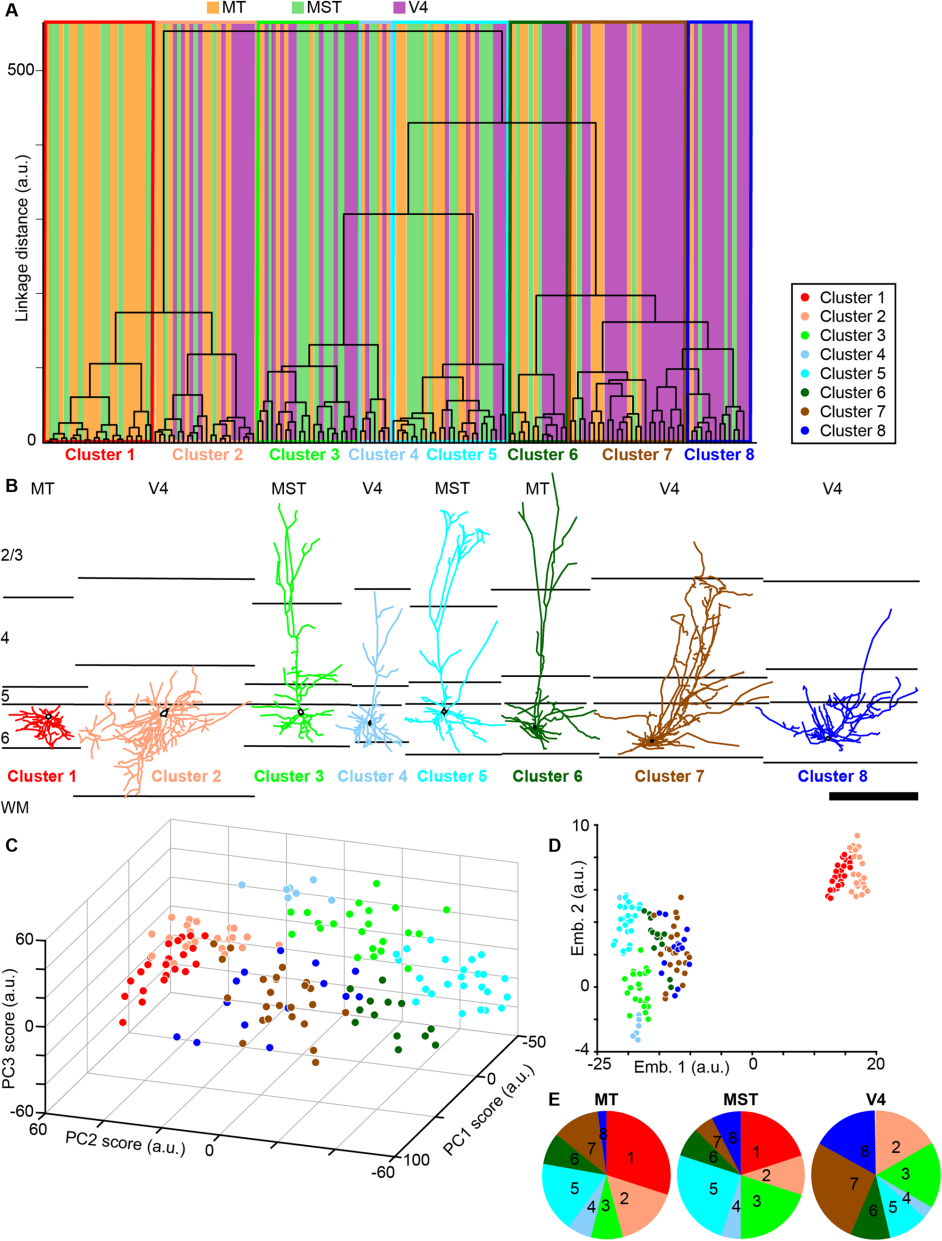
Clustering of extrastriate corticogeniculate neurons by morphological type and area of origin. ***A***, Dendrogram showing clustering of reconstructed extrastriate corticogeniculate neurons based on least-squared linkage distances between groups of neurons in an 11-dimensional space which represents each morphological metric (Tables 1–3). Colors indicate extrastriate area of origin of each corticogeniculate neuron: orange indicates MT, lime green indicates MST, and magenta indicates V4. Clusters are indicated by thin boxes with colored outlines representing cluster assignment (see legend). ***B***, Representative examples of reconstructed corticogeniculate neurons in each cluster (color-coded based on cluster assignment). These are the same reconstructions illustrated in Figure 1*A–H*; additional reconstructions per cluster are illustrated in Figure 3. Layers are indicated by black lines, labeled at left, and are aligned to the layer 5/6 border per reconstruction. Scale bar at right is 750 µm and applies to all reconstructions. Area of origin is listed above each reconstruction; cluster number is listed below. Cell bodies are indicated by black outlines. ***C***, Relationships between first, second, and third PC scores (arbitrary units) following PCA of the same 11 morphological metrics used in the cluster analysis; color coded from cluster analysis as in ***A*** and ***B***. ***D***, t-SNE plot generated from all PCA scores illustrating the first and second embeddings (arbitrary units); color coded from cluster analysis as in ***A*** and ***B***. ***E***, Pie charts illustrate the proportional distribution of extrastriate corticogeniculate neurons from each cluster within each area (cluster number indicated and clusters color-coded as in ***A***).

**Figure 3.**
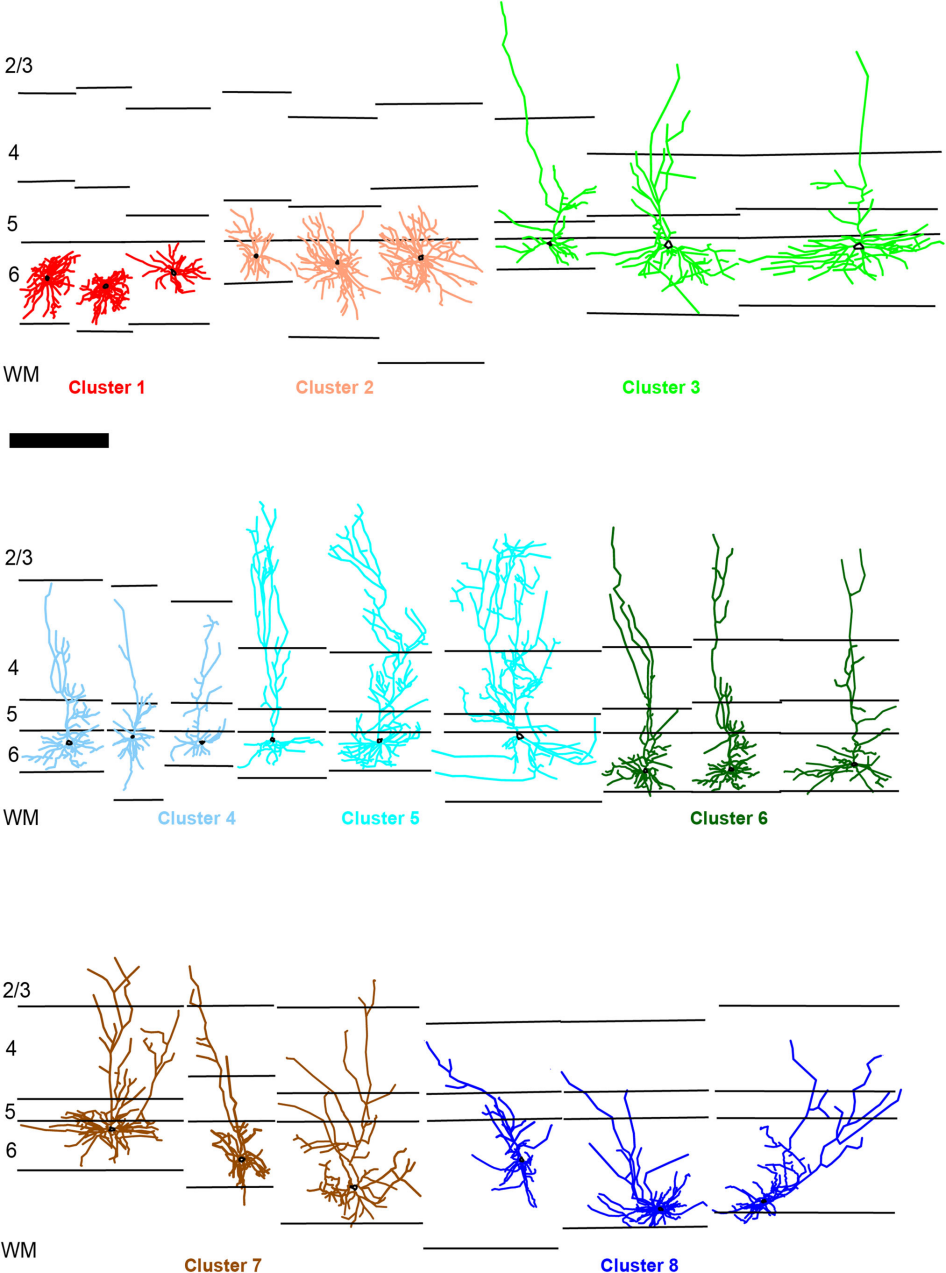
Corticogeniculate neurons per cluster. Additional examples of reconstructed corticogeniculate neurons in each cluster (color-coded based on cluster assignment as in Fig. 2 and labeled below each group). Layers are indicated by black lines, labeled at left, and are aligned to the layer 5/6 border per reconstruction. Scale bar at left is 750 µm and applies to all reconstructions. Cell bodies are indicated by black outlines.

To apply a more rigorous quantitative analysis of unique morphological cell types, we performed a cluster analysis on the full sample of 150 reconstructed extrastriate corticogeniculate neurons using 11 independent morphological metrics (listed in Tables 1–3; see Materials and Methods). Critically, no information about the area of origin of each extrastriate corticogeniculate neuron was included in the cluster analysis. The clustering algorithm weighted each morphological metric equally and clustered neurons based on least-squared distances between neurons in an 11-dimensional space. Two independent statistical tests of optimal cluster number, the Calinski/Harabasz method and GMMs, both revealed an optimal cluster number of 8. Interestingly, many of these clusters were dominated by corticogeniculate neurons originating in a single extrastriate area (Fig. 2*A*). Also, clusters contained neurons qualitatively identified as unique morphological types (Fig. 2*B*; note neurons illustrated in Fig. 1*A–H* are reproduced here for improved visibility of arbors, additional example reconstructions per cluster illustrated in Fig. 3). For example, Cluster 1 contained spiny stellate neurons mainly in MT while Cluster 8 contained short pyramidal neurons mainly in V4. A separate PCA performed on the same 11 morphological metrics further confirmed that neurons in the eight clusters identified in the cluster analyses could be differentiated based on multiple factors, as comparisons across the first three PC scores (Fig. 2*C*) and all PC scores (Fig. 2*D*) showed separation of neurons by cluster. Thus, through multiple independent quantitative methods, corticogeniculate neurons originating in areas MT, MST, and V4 clustered into eight morphologically distinct groups based on 11 morphological metrics and blind to area of origin.

Although most of the eight morphologically defined clusters contained at least some corticogeniculate neurons from each of the three areas (with the exception of Cluster 1 that only contained MT and MST neurons), area of origin was not uniformly distributed across clusters (Fig. 2*E*). This nonuniform distribution suggested correlations between morphological characteristics and area of origin. For example, Cluster 1 was dominated by MT neurons (15 of 23; Fig. 2*E*, red), all of which were spiny stellate cells (Fig. 2*B*, red). Cluster 2 also contained only spiny stellate cells (Fig. 2*B*, salmon), and included all of the V4 stellate cells (Fig. 2*E*, salmon). Cluster 3 (Fig. 2*B*, green) mostly contained standard tall cells, and a few standard short (3 of 22) and intermediate cells (2 of 22), most of which originated in MST and V4 (10 of 22 from V4 and 8 of 22 from MST compared with 4 of 25 from MT; Fig. 2*E*, green). Cluster 4 was composed mainly of standard short cells (and one intermediate cell; Fig. 2*B*, light blue), distributed across all three mid-level extrastriate areas (Fig. 2*E*, light blue). Cluster 5 was composed of only tall cells, most with apical dendritic tufts (20 of 25; Fig. 2*B*, cyan) and many of which were in MST (10 of 25; Fig. 2*E*, cyan). All cells in Cluster 6 had tall apical dendrites, some with apical dendritic tufts (5 of 13; Fig. 2*B*, green), but these were more distributed across all three mid-level extrastriate areas (Fig. 2*E*, dark green). Cluster 7 differed from the other clusters in that it contained multiple qualitatively defined cell types. However, most of the corticogeniculate neurons in Cluster 7 were similar to the recently identified “intermediate” corticogeniculate cells (Adusei et al., 2021) with apical dendrite height intermediate to that of standard tall and short pyramidal corticogeniculate neurons (9 of 24; Fig. 2*B*, brown). Also, most of the cells in Cluster 7 were located in V4 (16 of 24; Fig. 2*E*, brown). While many of the cells in Cluster 8 were short cells (7 of 14), this cluster was unique in that it contained of a group of corticogeniculate neurons called “tilted” cells (Briggs et al., 2016). Tilted cells are characterized by a marked tilt in the angle at which the base of the apical dendrite exits the cell body (Fig. 2*B*, blue); that is, the apical dendrite leaves the cell body from the side rather than the top, as is common for most pyramidal-shaped neurons. In line with area-specific clustering, most of the tilted cells in Cluster 8 were located in V4 (Fig. 2*E*, blue).

**Figure 4.**
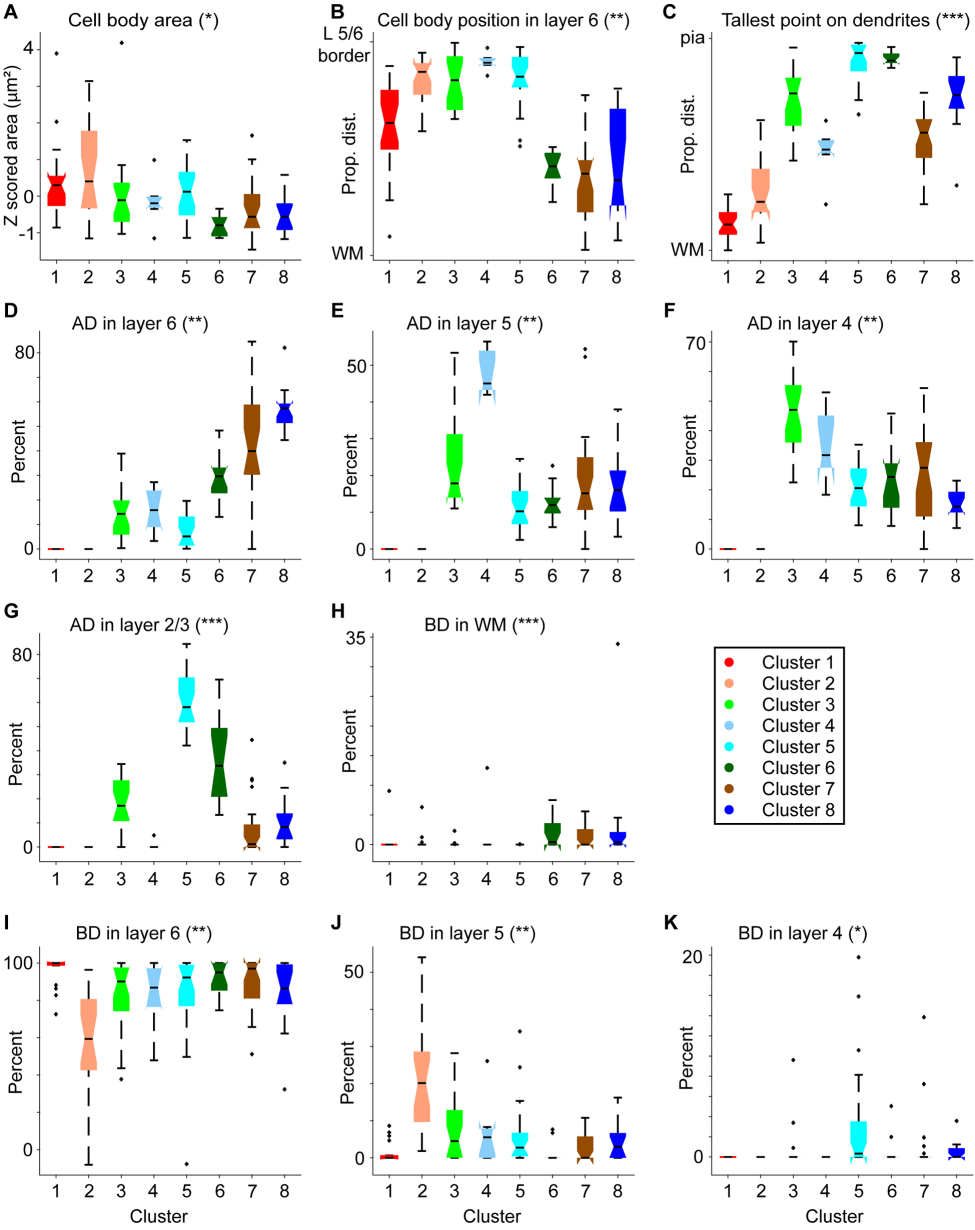
Quantitative assessment of morphological metrics for each cluster of extrastriate corticogeniculate neurons. Clusters are color coded according to cluster assignment (as in Figs. 1, 2; see legend at right). Notched box and whisker plots illustrate medians (horizontal black lines), 25th and 75th percentiles (notched boxes), data distributions (black lines above, below boxes), and outliers (black crosses). Asterisks with panel titles indicate the number of comparisons across clusters that were statistically significant (i.e., * = 1 or more significant differences between clusters, ** = 2+ statistically significant differences between clusters, etc.). See Tables 1–3 for all statistics. ***A***, Cell body area, *z*-scored. ***B***, Cell body position as percent depth in layer 6; WM indicates the white matter boundary. ***C***, Height of the apical dendrite or tallest point on basal dendrite for spiny stellate cells. ***D–G***, Percentage of apical dendrite (AD) in layers 6 (***D***), 5 (***E***), 4 (***F***), and 2/3 (***G***). ***H–K***, Percentage of basal dendrite (BD) in WM (***H***), and in layers 6 (***I***), 5 (***J***), 4 (***K***).

After confirming nonuniform distributions of corticogeniculate neurons from MT, MST, and V4 per cluster, we next quantified the morphological characteristics that defined each cluster. Certain morphological features contributed to robust clustering, and often these features were common to neurons originating in different areas. Cell body size contributed to clustering in a manner correlated with cell body position in layer 6. Neurons in Clusters 1, 2, 3, and 5 had significantly larger cell bodies compared with Cluster 6 neurons (Fig. 4*A*, Table 1) and were generally positioned in the upper portion of layer 6. Cell bodies of neurons in Clusters 2, 3, and 5 were significantly more superficial in layer 6 relative to neurons in Clusters 6, 7, and 8, whose cell bodies were positioned deep in layer 6, closer to the white matter (Fig. 4*B*, Table 1). Similarly, standard short corticogeniculate neurons that made up Cluster 4 were located more superficially relative to neurons in Clusters 6, 7, and 8 (Fig. 4*B*, Table 1) and tended to also have larger cell bodies (Fig. 4*A*, Table 1), although this latter difference was not significant.

Apical dendrite height and proportions of apical dendrite per layer also defined clusters. Since spiny stellate cells lack apical dendrites, it was not surprising that the tallest points on the dendritic trees of corticogeniculate neurons in Clusters 1 and 2, all of which were spiny stellate cells, were significantly further from the pial surface compared with those of neurons in Clusters 3, 5, 6, and 8, which included tall/tufted and tilted cells (Fig. 4*C*, Table 1). Short and intermediate neurons in Clusters 4 and 7 also had more superficial dendrites compared with neurons in Clusters 1 and 2, although only Clusters 1 and 7 differed significantly in dendritic height (Fig. 4*C*, Table 1). Of the clusters containing nonstellate corticogeniculate neurons, tall/tufted pyramidal cells in Clusters 3, 5, and 6 had the tallest apical dendrites, followed by tilted (Cluster 8) and intermediate (Cluster 7) cells, followed by standard short cells in Cluster 4 (Fig. 4*C*, Table 1). Accordingly, significant differences in apical dendritic height were observed between the tallest corticogeniculate neurons in Clusters 5 and 6 and the short and intermediate neurons in Clusters 4 and 7, respectively (Table 1).

As with dendritic height, differences in proportions of apical dendrite per layer across clusters were driven by the fact that clusters containing spiny stellate neurons (Clusters 1 and 2) had no apical dendrites (Fig. 4*D–G*, Table 2). However, clusters containing nonstellate neurons were also differentiated by the proportion of apical dendrite in each layer. Tall/tufted pyramidal corticogeniculate neurons in Clusters 5 and 6 had higher proportions of apical dendrites in layer 2/3 compared with all other clusters (reaching significance for all comparisons except between Cluster 6 and Clusters 3 and 8; Fig. 4*G*, Table 2). Importantly, this significantly separated tall/tufted corticogeniculate neurons (Clusters 5 and 6) from short and intermediate neurons (Clusters 4 and 7). Cluster 3 also contained tall corticogeniculate neurons, but these had significantly less apical dendrites in layer 2/3 and significantly more apical dendrites in layer 4 compared with tall/tufted corticogeniculate neurons in Cluster 5 (Fig. 4*F,G*, Table 2). As expected, based on their deeper cell body positions, neurons in Clusters 6, 7, and 8 had greater proportions of apical dendrite in layer 6 (Fig. 4*D*, Table 2). Interestingly, most corticogeniculate neurons had relatively low proportions of apical dendrite in layer 5, with the notable exception of standard short neurons in Cluster 4, which had significantly more apical dendrites in layer 5 compared with nonstellate neurons in Clusters 5 and 6 (Fig. 4*E*, Table 2).

The vast majority (∼80–98%) of basal dendrites for all corticogeniculate neurons were in layer 6, with very small proportions of basal dendrites encroaching into layers 5 and 4 or the white matter (Fig. 4*H–K*, Table 3). Nonetheless, percentage of basal dendrite per layer or white matter also contributed to clustering. Importantly, proportions of basal dendrite in layers 5 and 6 significantly differentiated spiny stellate cells in Clusters 1 and 2 (Table 3). Due to their more superficial cell body positions in layer 6, spiny stellate cells in Cluster 2 all had basal dendrites in layer 5, whereas few (6 of 23) spiny stellate cells in Cluster 1 had dendrites in layer 5 (Fig. 4*J*, Table 3). Consequently, Cluster 1 spiny stellate cells had significantly more basal dendrites in layer 6 compared with those in Cluster 2 (Fig. 4*I*, Table 3). It is also noteworthy that corticogeniculate neurons in Cluster 2 had significantly more basal dendrites in layer 5 compared with all the other corticogeniculate clusters (except Cluster 4), perhaps suggesting unique local circuit relationships for this group of spiny stellate cells. Not surprisingly, proportions of basal dendrites per layer correlated with cell body position in layer 6 for nonstellate corticogeniculate neurons as well. Superficially positioned neurons in Cluster 5 had more basal dendrites in layer 4 and no basal dendrites in the white matter (Fig. 4*H*,*K*; Table 3). Along the same lines, corticogeniculate neurons with cell bodies deep in layer 6 (Clusters 6, 7, and 8) all had some proportion of basal dendrite in the white matter (Fig. 4*H*, Table 3). Deep cell body position and dendrites in the white matter is a hallmark of tilted corticogeniculate neurons described previously (Briggs et al., 2016; Hasse et al., 2019; Adusei et al., 2021) and also observed here among tilted cells in Cluster 8.

The cluster analysis thus separated corticogeniculate neurons into distinct subpopulations from those qualitatively identified. For example, Clusters 1 and 2 were made up entirely of spiny stellate cells, but neurons across these clusters differed significantly in their cell body positions within layer 6, which correlated with different distributions of basal dendrites in layers 5 and 6 across clusters. Clusters 3, 5, and 6 all contained tall and tall/tufted corticogeniculate neurons. But these differed in their cell body positions in layer 6 (superficial for Clusters 3 and 5, deep for Cluster 6) as well as their proportions of apical dendrites in layers 4 and 2/3. Clusters 4, 7, and 8 contained neurons with shorter apical dendrites (compared with tall/tufted clusters), but each of these clusters was distinct in a number of ways. Cluster 4 was made up of standard short corticogeniculate neurons with cell bodies positioned at the top of layer 6 and a large proportion of apical dendrite in layer 5 and none in layer 2/3. Cluster 7 neurons were intermediate but always located deep in layer 6 and with a greater proportion of apical dendrite in layer 6. Finally, Cluster 8 contained the unusual tilted corticogeniculate cell type, also with cell bodies at the bottom of layer 6 and thus a large proportion of apical dendrite in layer 6, but also with taller apical dendrites and some amount of dendrite in layer 2/3. The presence of distinct classes of corticogeniculate neurons, each with unique distributions of dendrites spanning the cortical layers, supports the notion that each class receives different patterns of local cortical inputs and may therefore relay unique signals back to the LGN.

## Discussion

Here we provide the first morphological description of corticogeniculate neurons in mid-level extrastriate cortical areas of the primate brain. Corticogeniculate neurons have been observed in V2 in primates (Lin and Kaas, 1977; Briggs et al., 2016) and tritiated proline injections into extrastriate cortex yielded small amounts of label in the LGN, but whether this label was transported directly from extrastriate cortex or trans-synaptically was not known (Lin and Kaas, 1977; Hendrickson et al., 1978). Here we demonstrate that corticogeniculate neurons are sparse but consistently present in mid-level extrastriate visual cortex of the primate. Even though the proportion of corticogeniculate neurons is likely to decrease by a factor of 1–2 orders of magnitude at each stage of the cortical processing hierarchy, it is noteworthy that virus-infected corticogeniculate neurons were observed in every section of mid-level extrastriate cortex examined, even when virus injections into the LGN were of small volumes. Furthermore, extrastriate corticogeniculate neurons were located throughout MT, MST, and V4, suggesting they are not restricted to central or peripheral visual representations. Additionally, extrastriate corticogeniculate neurons included at least eight unique morphologically defined cell types, although these cell types were not uniformly represented across the three areas. Instead, specific morphological subtypes were predominantly located in single cortical areas (e.g., spiny stellate neurons with cell bodies in lower layer 6 were mainly in MT). The vast majority of corticogeniculate neurons in MT and MST had spiny stellate morphology, which could suggest putative relationships with the koniocellular layers of the LGN, as discussed below. However, it is also important to note that tall, short, intermediate, and tilted corticogeniculate neurons were also observed across all three mid-level extrastriate areas, providing a possible substrate for parallel channels of extrastriate corticogeniculate feedback to the LGN.

The virus-mediated retrograde circuit tracing method (Wickersham et al., 2007; Osakada et al., 2011) has proven highly effective for visualizing less common and unusual neuronal types compared with more traditional retrograde tracing techniques. To prevent any biases in our reconstruction analyses, we reconstructed neurons that were separated from their neighbors (90 of 150 total reconstructed neurons), as well as those that were within denser clusters of labeled neurons (60 of 150 total reconstructed neurons). Since 40% of our reconstructions came from denser clusters, it is likely that the relative proportions of qualitatively identified cell types that were included in the cluster analysis may not be representative of the true proportions of morphologically unique cell types in extrastriate cortex. To resolve this bias, we also manually counted qualitatively identified corticogeniculate neurons in MT, MST, and V4—this was feasible given the lower density of virus-infected corticogeniculate neurons in extrastriate cortex compared with V1 and V2. It is from these manual counts that we determined that spiny stellate corticogeniculate neurons are highly overrepresented in MT and MST and also make up the majority of corticogeniculate neurons in V4. In qualitatively assigning cell type to manually counted corticogeniculate neurons, we may also introduce some bias, but we do not believe this altered the main finding that spiny stellate corticogeniculate neurons are significantly overrepresented in extrastriate cortex. Furthermore, the cluster analysis separated corticogeniculate neurons into groups that largely matched our qualitative assignments.

### Evidence supporting parallel streams of corticogeniculate feedback to LGN

Multiple independent lines of research converge in support of the functional organization of corticogeniculate neurons into parallel processing streams. In primate V1, the cell bodies of corticogeniculate neurons are restricted to the upper and lower thirds of layer 6, reflecting the distribution of parvocellular and magnocellular geniculocortical axonal collaterals that target these layer 6 subdivisions, respectively (Lund et al., 1975; Conley and Raczkowski, 1990; Fitzpatrick et al., 1994; Ichida and Casagrande, 2002). Cell bodies of corticogeniculate neurons that project to parvocellular LGN layers reside in the top tier of layer 6 and those that project to the magnocellular LGN layers have cell bodies in the bottom tier of layer 6, while koniocellular-projecting corticogeniculate neurons likely originate from upper and lower tiers of layer 6 (Fitzpatrick et al., 1994). These findings combined with more detailed morphological characterization of V1 corticogeniculate neurons suggest that standard short corticogeniculate cells with cell bodies in the top tier of layer 6 project to parvocellular LGN layers while standard tall corticogeniculate cells found in the bottom tier of layer 6 project to magnocellular LGN layers. Additional and less common morphological cell types, including spiny stellate, tilted, and tall-tufted cells, most likely project to the various koniocellular LGN layers (Briggs et al., 2016).

Further evidence in favor of a parallel functional organization of V1 corticogeniculate neurons comes from physiological measures demonstrating that visual physiological response properties correlate with axon conduction speed in primates and carnivores (Harvey, 1978; Tsumoto and Suda, 1980; Grieve and Sillito, 1995; Briggs and Usrey, 2005, 2007). Furthermore, in primates, fast-conducting complex corticogeniculate cells had visual response properties consistent with magnocellular LGN neurons; medium-conducting simple cells had responses consistent with parvocellular LGN neurons; and slowly conducting complex cells had responses consistent with koniocellular LGN neurons (Briggs and Usrey, 2009). These data together with laminar and morphological characterizations suggest that standard short, medium-conducting simple cells target parvocellular LGN neurons; standard tall, fast-conducting complex cells target magnocellular LGN neurons; and spiny stellate, tilted, and other unusual corticogeniculate cell types with slowly conducting axons target koniocellular LGN neurons.

Homologies in corticogeniculate morphological types across species and visual areas (Briggs et al., 2016; Hasse et al., 2019; Adusei et al., 2021) support parallel functional organization of corticogeniculate feedback throughout the visual system. In other words, the presence of heterogeneous corticogeniculate cell types across all visual areas studied to date suggests that multiple distinct corticogeniculate cell types per area is the rule in primates and carnivores. Under this scheme, standard short and standard tall extrastriate corticogeniculate neurons may target the parvocellular and magnocellular LGN layers, respectively, while the other cell types, including spiny stellate cells, may target koniocellular layers. It is interesting to note that while V1 contains a smaller proportion of spiny stellate neurons, these make up the majority of corticogeniculate neurons in mid-level extrastriate cortex. The fact that spiny stellate cells are the majority of corticogeniculate cells in MT is consistent with results showing that the majority of MT-projecting geniculocortical cells originate in the koniocellular layers and are labeled with CamKII, a marker for koniocellular neurons (Sincich et al., 2004). Together, these results support a segregation of feedforward and feedback circuits based on functional stream identity.

Acknowledging limitations in functional predictions based purely on anatomical data, our results at a minimum suggest that the distinct classes of corticogeniculate neurons in extrastriate cortex receive unique patterns of local cortical inputs. This is based on the different distributions of dendrites across cortical layers that contributed to defining the eight clusters we observed. In particular, two different groups of spiny stellate neurons were identified, separated in part by differential distributions of basal dendrites in layers 5 and 6. Given that neurons in layers 5 and 6 are likely to have diverse functional properties (Kritzer et al., 1992; Levitt et al., 1994), it is reasonable to predict that these two types of spiny stellate neurons integrate distinct types of visual information. It is also noteworthy that one of these clusters contained mostly MT neurons while the other cluster contained a mixture of MT, MST, and V4 spiny stellates. Thus, regardless of whether or not spiny stellate corticogeniculate neurons target koniocellular LGN layers, it is likely that they convey visual signals to the LGN that differ from those of other corticogeniculate cell types and even perhaps from other spiny stellate sub-classes.

While MT and MST were heavily dominated by spiny stellate corticogeniculate neurons, V4 contained a more balanced representation of different morphological types. Again, following the logic laid out for V1 corticogeniculate neurons, this could suggest that feedback to the LGN from V4 targets parvocellular, magnocellular, and koniocellular layers of the LGN in more equivalent proportions. A study of geniculocortical neurons projecting directly to V4 found that while many stained for koniocellular markers, others did not (Rodman et al., 2001), suggesting a broader distribution of LGN types projecting to V4. There are several possible explanations for differential connectivity between V4 and LGN compared with that from MT and MST, including the fact that V4, a part of the ventral stream, is a significantly larger cortical area containing a greater number of neurons overall. It is notable that Clusters 7 and 8, containing mainly intermediate and tilted corticogeniculate neurons, were dominated by neurons from V4. Similarly, no V4 spiny stellates were in Cluster 1 (all V4 spiny stellate neurons were in Cluster 2). The fact that these areal biases emerged from the morphological cluster analysis suggests that corticogeniculate circuits originating in the ventral stream (V4) are distinct from those originating in the dorsal stream (MT and MST). It would be very interesting to discover how V4 versus MT/MST corticogeniculate neurons connect with LGN neurons in the magnocellular/parvocellular/koniocellular layers. Are inputs from ventral and dorsal streams kept apart within the LGN? Or are LGN neurons integrating information from across the dorsal and ventral streams, serving more as a visual information hub?

In order for the LGN to serve as a hub for integrating visual information across the processing hierarchy, bidirectional connections (i.e., feedforward/feedback loops) should link LGN directly with multiple visual cortical areas. Given that direct feedforward geniculocortical connections to MT and V4 have been described previously (Yukie and Iwai, 1981; Bullier and Kennedy, 1983; Cowey and Stoerig, 1989; Rodman et al., 2001; Sincich et al., 2004), our findings presented here effectively “close the loop.” It is not known whether individual corticogeniculate neurons in MT, MST, or V4 themselves receive geniculocortical inputs, as has been shown for a subset of V1 corticogeniculate neurons (Briggs and Usrey, 2007). However, poly-synaptic geniculo-cortico-geniculate circuits connecting LGN with extrastriate cortex would nonetheless allow the LGN to integrate higher-order visual signals from extrastriate cortex.

As indicated above, it is important to use caution in making functional claims regarding cell type distinctions based only on morphological data. Without functional or visual physiological data from extrastriate corticogeniculate neurons, we cannot rule out the possibility that corticogeniculate neurons in MT, MST, and V4 follow a different set of rules regarding their connectivity with the LGN. However, participation in distinct local circuits within each area, as dictated by different distributions of dendrites across corticogeniculate types, suggests some functional specialization across cell types.

### Functional implications of extrastriate corticogeniculate circuits

Parallel streams of corticogeniculate feedback from multiple extrastriate cortical areas could form a series of nested geniculo-cortico-geniculate loops supporting a role for the LGN as a hub for visual information sampled at multiple stages along the visual processing hierarchy. These nested loops could improve response precision by reducing variability among LGN neurons, as demonstrated for corticogeniculate feedback from V1 (Hasse and Briggs, 2017b; Murphy et al., 2021). Although extrastriate corticogeniculate circuits are sparse, their consistent presence throughout the visual cortex suggests preservation through evolution of the primate visual system and a critical role in some aspects of visual function. Additionally, extrastriate geniculo-cortico-geniculate loops may be critical for residual vision following V1 damage. In a primate model of blindsight, the LGN, and by extension extrastriate geniculo-cortico-geniculate loops, were necessary for visual stimulus detection in the blind field (Schmid et al., 2010). Evidence from human patients with V1 lesions also suggests that residual visual capabilities correlate with connectivity between the LGN and MT (Ajina et al., 2015; Tamietto and Morrone, 2016), again suggesting dependence upon extrastriate geniculo-cortico-geniculate loops. It is also possible that extrastriate corticogeniculate feedback is critical in remodeling of geniculo-cortico-geniculate circuits that has been shown to occur following V1 lesions (Atapour et al., 2022).

The consistent presence of corticogeniculate neurons throughout the visual cortex suggests that other sensory systems may contain corticothalamic neurons beyond secondary sensory cortex that also target first-order thalamus. Indeed, broader characterizations of these sparse but consistently present circuits could provide additional clues about the overall functional roles of corticothalamic feedback in sensory perception.

## Synthesis

Reviewing Editor: Nicholas J. Priebe, University of Texas at Austin Decisions are customarily a result of the Reviewing Editor and the peer reviewers coming together and discussing their recommendations until a consensus is reached. When revisions are invited, a fact-based synthesis statement explaining their decision and outlining what is needed to prepare a revision will be listed below. The following reviewer(s) agreed to reveal their identity: NONE. Note: If this manuscript was transferred from JNeurosci and a decision was made to accept the manuscript without peer review, a brief statement to this effect will instead be what is listed below.

SYNTHESIS

Two reviewers evaluated your revised manuscript and appreciated the changes that had been made.

There remain only a few issues of clarification.

1) Neurons that appear similar

There are some reconstuctions that are very similar to each other. A specific example is given below. These similarities may be due to common patterns that emerge across neurons or to the Neurolucida sampling resolution. As written in the previous review, the authors should include a comment in the text about why such branching pattens might be common in reconstructions, even though the neurons are not the same.

2) Laminar determination

Please add some description of layer 4 and 5 border determinations (somewhere in lines 220‐230), as it influences all the quantitation and the borders are very difficult to see in this histological material. Given their comparison with Nissl stained sections (and the table provided in the rebuttal), it seems like a good idea to incorporate a description of the careful approach for layer assignments into the Methods section, as it would bolster confidence in the layer assignments. The current version of the manuscript does not mention any of this Nissl comparison.

3) Clusters from PCA data

Unfortunately the 3D plot of PCA data in Fig. 2C still does not give a reader much confidence in there being 8 clusters, likely because higher dimensions in the data (there are 11) presumably pull the clusters apart. Is there a way to display, perhaps, another panel showing three of the higher PCA components (e.g. 4‐6) for the data where the clusters are more visibly separated? Would a tSNE plot work? We understand that the statistical analysis says there are 8 clusters, and this may be hard to display. In the end, the addition of new Fig. 3, with 3 more cells in each category, was a sound choice and appears convincing. But there is no call out in the text to this new Fig. 3 (this should happen around line 400).

## Author Response

We thank the editors and the reviewer for their continued scrutiny of our work, including the raw data files that we provided. We are happy to address the remaining concerns with revisions to the manuscript text and figures as recommended. Changes to the manuscript are indicated as red text and point-by-point responses to each concern are below.

Concern 1) Neurons that appear similar There are some reconstructions that are very similar to each other. A specific example is given below. These similarities may be due to common patterns that emerge across neurons or to the Neurolucida sampling resolution. As written in the previous review, the authors should include a comment in the text about why such branching pattens might be common in reconstructions, even though the neurons are not the same.

Response 1: As recommended, we have added a sentence to the latter part of the third paragraph of Results: "Importantly, neurons that were qualitatively similar shared some dendritic features, like branched apical dendrites at similar laminar locations or extending basal dendrites with similar shapes, suggesting that extrastriate corticogeniculate neurons could form discrete groupings or clusters, explored further below." The fact that a few neurons share similar apical dendritic features and a few share similar basal dendritic features (examples in A of the reviewer's comments, i.e., reviewer's figures A and B) across a sample of 150 neurons is perhaps not surprising. Furthermore, these similarities support our overall conclusion that extrastriate corticogeniculate neurons can be clustered into discrete groups based on similarities in dendritic morphology. These similarities are not a result of resolution limitations in the Neurolucida system (which has micron-scale resolution). To the reviewer's point B, regarding variations across each submission version, we believe we have addressed this in detail in each of our prior responses. But to reiterate, we fully revisited all neurons in the second revision, including adding to branches through more adjacent sections and also ensuring that we did not include any branches that we could not unambiguously attribute to the reconstructed neuron (this thorough revision of all reconstructions meant neurons were grouped into different clusters - noted again by the reviewer in their figure E). We further revisited all neurons highlighted by the reviewer in the third version, as requested. This is why some reconstructions have more branches across versions and some have fewer. We have highlighted the text of the Methods (top of fourth paragraph) that describes reconstructions only of branches unambiguously attached to each parent neuron.

2) Laminar determination Please add some description of layer 4 and 5 border determinations (somewhere in lines 220‐230), as it influences all the quantitation and the borders are very difficult to see in this histological material. Given their comparison with Nissl stained sections (and the table provided in the rebuttal), it seems like a good idea to incorporate a description of the careful approach for layer assignments into the Methods section, as it would bolster confidence in the layer assignments. The current version of the manuscript does not mention any of this Nissl comparison.

Response 2: We added the following text to the Methods (middle of third paragraph): "Because the boundaries between layers could be difficult to discern from cytochrome oxidase staining in some of extrastriate cortex, we computed the relative depths of each cortical layer in MT, MST, and V4 based on our cytochrome oxidase-stained tissue and compared that with depths of each layer per area from Nissl-stained tissue (from Brainmaps.org and U. Rochester Neuroscience Department anatomical archive). In all cases, our cytochrome oxidase-based laminar depths were within 2.5% of the laminar depths determined from Nissl-stained tissue." 3) Clusters from PCA data Unfortunately the 3D plot of PCA data in Fig. 2C still does not give a reader much confidence in there being 8 clusters, likely because higher dimensions in the data (there are 11) presumably pull the clusters apart. Is there a way to display, perhaps, another panel showing three of the higher PCA components (e.g. 4‐6) for the data where the clusters are more visibly separated? Would a tSNE plot work? We understand that the statistical analysis says there are 8 clusters, and this may be hard to display. In the end, the addition of new Fig. 3, with 3 more cells in each category, was a sound choice and is convincing. But there is no call out in the text to this new Fig. 3 (this should happen around line 400).

Response 3: We have added a tSNE plot to Figure 2 (new Figure 2D) to further show the separation of the 8 clusters.

Figure 3 is called out in the Results, middle of the fourth paragraph, now shown in red text.
